# Supplementary material for: Weighted Geometric Mean (WGM) method: A new chromatic adaptation model
Source: PLoS One. 2023 Aug 14;18(8):e0290017. doi: 10.1371/journal.pone.0290017 (PMC10424855; doi:10.1371/journal.pone.0290017)
Supplement: Attachment — Submitted filename: Response to Reviewers.docx [file pone.0290017.s001.docx]

Dear Editor,

Thank you for considering our manuscript **Weighted Geometric Mean (WGM) method: A new chromatic adaptation model** for publication in PLOS ONE. We appreciate the time and effort that you and the reviewers have taken to provide valuable feedback on our work.

We have carefully considered the points raised by the academic editor and reviewer(s) and would like to respond to each of them as follows:

**From reviewer(s):**

**Point 1:  I think a couple of more sentences in the abstract can be helpful for contextualizing the problem better.**

Our Response: Some sentences added in the abstract.

**Point 2: Please include Von Kries reference on line 25 of page 1.**

Our Response: Included

**Point 3: The D value in the adaptation has been set by optimization. Please detail what dataset has been used to do this and whether the found value is good for other datasets.**

Our Response: The D- value is highly depended on various view condition such as ambient light intensity and correlated color temperature (CCT), etc. Due to the absence of robust model in predicting D value, we choose to optimize it to best fit the experiment data for each model. Therefore, comparing each CAT model is only related to model itself rather than the method setting D value.

**Point 4: Can D be set just this way of is setting D an open problem?**

Our Response: As the mentioned in point 3, this D-value setting method enables us to compare model performance instead of the methodology in setting D value, which remains an open problem in color science arear. The equation used to set the D-value in the widely-used CAT model, CAT16, only takes into account the ambient light intensity and surround condition. However, there may be other factors that could potentially affect D-value such as the chromaticity value of ambient light.

Here are some references in discussing setting D:

Smet, Kevin AG, et al. "Study of chromatic adaptation using memory color matches, Part II: colored illuminants." *Optics express* 25.7 (2017): 8350-8365.

Zhai, Qiyan, and Ming R. Luo. "Study of chromatic adaptation via neutral white matches on different viewing media." *Optics express* 26.6 (2018): 7724-7739.

**Point 5: Include observer's responses variability in table 3 for each dataset. This is important to contextualize the differences between the methods to the observer's variability, which is helpful in understanding how different the methods perform.**

Our Response: Included the violin plot as figure 8 which shows the observer response variability in table 3 for each dataset.

**Point 6:  If there was a way to make statistical tests taking into account observers variablity, it would be great.**

Our Response: Thanks for the comments. However, there might not be a good way to make the statistical test since some datasets are only based on few observers' data (Fairchild reversibility and Shen data) while Derby/Leeds data only shows the observer's average results.

We have made the necessary revisions and amendments to our manuscript based on the comments and suggestions provided. We hope that our responses and revisions address all concerns and criticisms raised and demonstrate our commitment to improving the quality and clarity of our research.

Thank you again for your time and consideration.

Sincerely

Che Shen and Mark Fairchild
